# Editorial: Tick-borne viruses of domestic livestock: Epidemiology, evolutionary trends, biology and climate change impact

**DOI:** 10.3389/fvets.2023.1147770

**Published:** 2023-03-21

**Authors:** Daniel O. Oluwayelu, Sara Moutailler, Solomon O. Odemuyiwa

**Affiliations:** ^1^Department of Veterinary Microbiology, University of Ibadan, Ibadan, Nigeria; ^2^Centre for Control and Prevention of Zoonoses (CCPZ), University of Ibadan, Ibadan, Nigeria; ^3^ANSES, INRAE, Ecole Nationale Vétérinaire d'Alfort, UMR BIPAR, Laboratoire de Santé Animale, Maisons-Alfort, France; ^4^Department of Veterinary Pathobiology, College of Veterinary Medicine, University of Missouri, Columbia, MO, United States

**Keywords:** arboviruses, ticks, livestock, epidemiology, climate change

Tick-borne viral diseases (TBVDs) of domestic livestock pose severe threats to global food security, economies of nations and public health due to their negative consequences on farmers' incomes as well as human and animal health (1, 2). Adequate knowledge of the epidemiology of these diseases, and the adaptation mechanisms of ticks and the viruses they transmit to domestic animals coupled with the effect of climate change on incidence and geographical spread of the diseases is key to achieving universal food security and health (3, 4). Our Research Topic is focused on showcasing high-quality publications on TBVDs that have considerable impact on the health of domestic livestock and humans with a focus on viral emergence and tick-human interactions.

The increasing incidence and geographical distribution of tick-borne viruses underscore the animal and public health importance of these arboviruses, especially with the rapidly changing global climatic conditions. In a study conducted in Romania, one of the most biogeographically diverse countries in Europe with conducive environment for the establishment of tick-borne virus foci (5), Bratuleanu et al. reported the detection of novel viral sequences in *Rhipicephalus sanguineus* ticks collected from small ruminants. These novel viruses belonged to the *Phenuiviridae* (Brown dog tick phlebovirus 1 and Brown dog tick phlebovirus 2) and *Chuviridae* (Cataloi mivirus) families. Surprisingly, sequences from a novel quaranjavirus-like virus, tentatively called Cataloi tick virus, were also identified in these hard ticks. The authors concluded that the discovery and characterization of tick-transmitted viruses would have consequences for virus taxonomy and provide better understanding of tick-borne viral zoonoses.

The re-emergence and unceasing spread of recognized TBVDs such as Crimean-Congo hemorrhagic fever virus (CCHFV) positively correlate with the rising cases of TBVDs in humans and animals (Qin et al.). However, the emergence of some unknown TBVDs pathogens and the lack of data on tick virome underscores a crucial need for active viral surveillance and discovery in ticks, thereby promoting knowledge on the biodiversity and evolution of tick-transmitted viruses (Qin et al.). To gain better understanding of the spectrum of viruses borne by ticks and detect the likely viral pathogens, profiling of viruses in ticks is necessary. Using metagenomics sequencing, Qin et al. characterized the virome in three major tick species (*Haemaphysalis concinna, Dermacentor silvarum*, and *Ixodes persulcatus*) associated with pathogens in North China. They identified 28 RNA viruses belonging to more than 12 viral families of which Dermacentor pestivirus-like virus, Chimay-like rhabdovirus, taiga tick nigecruvirus, and Mukawa virus were recognized as novel viruses. Additionally, they detected Nuomin virus, Scapularis ixovirus, Sara tick-borne phlebovirus, Tacheng uukuvirus, and Beiji orthonairovirus which are already recognized as human pathogens with unknown geographical distribution and pathogenicity.

Crimean-Congo hemorrhagic fever virus is maintained in nature *via* ticks and animals. Ticks, mainly from the *Hyalomma* genus, are considered biological vectors and reservoirs of the virus (6). This virus causes hemorrhagic fevers in humans while it is asymptomatic in animals (7). Despite this, animals are the source of infection for ticks and can be the source of human infection *via* contaminated body fluids (8). The disease is considered emerging in Europe and the Mediterranean Basin with recurrent epidemics in some countries (as in Turkey), and an increase in the number of human cases in others (as in Spain and Greece) (9, 10). In France (mainland and Corsica), where the tick *Hyalomma marginatum* is present, seroprevalence studies have shown the presence of anti-CCHFV antibodies in animals (11, 12), suggesting that CCHFV could be spreading silently (indeed no human cases reported). Bernard et al. in their review (based on the analysis of 1,035 articles), studied the epidemiology of CCHF at the tick-domestic livestock-ecosystem interface in France and identified different factors that could explain the prevailing situation.

Contrary to its current epidemiology in Europe, CCHF is an endemic disease in the Arab world, a term that describes a large geographically heterogeneous area that covers the northern part of Africa and extends to the Levant. In their systematic review, Perveen and Khan examined the epidemiology of CCHF in the Arab world from 1978 to 2021. Although the review documented widespread infection of several animal species as evidenced by serological assays, CCHF was most frequently only recognized following fatal human infections. Like Europe, *Hyalomma* ticks appeared to be the most common vector of CCHF in the Arab world; however, the role of *Rhipicephalus* species has not been extensively investigated. Most importantly, a consensus of all the 57 studies examined is that CCHF is an occupational hazard among workers in the livestock processing industry. The review highlighted the need for a more focused surveillance activity to describe the epidemiology of CCHF in the Arab world. This call is apt at a time when many tick vectors are rapidly expanding their geographic ranges due to changes in global climatic conditions. Otherwise, an endemic viral infection in the Arab world could soon become an epidemic in Europe and the rest of the world.

The results of these studies and reviews not only provide new relevant information on the extensive diversity of RNA viruses in ticks, but they also offer a basis for further studies on the interactions among domestic livestock, ticks and tick-borne viruses. Besides, they provide better awareness of tick-borne viruses and the possible threat they constitute to ruminants and humans. They are also valuable for preventing possible outbreaks of TBVDs of livestock through vector control. Additional studies to elucidate the geographical distribution, infectivity, and pathogenicity of these tick-borne viruses are required.

## Author contributions

DO wrote the introduction and conclusion, while all authors contributed to the central sections with comments to the cited papers and references. All authors read and approved the submitted version.
